# Allosteric Regulation of Fibronectin/α_5_β_1_ Interaction by Fibronectin-Binding MSCRAMMs

**DOI:** 10.1371/journal.pone.0159118

**Published:** 2016-07-19

**Authors:** Xiaowen Liang, Brandon L. Garcia, Livia Visai, Sabitha Prabhakaran, Nicola A. G. Meenan, Jennifer R. Potts, Martin J. Humphries, Magnus Höök

**Affiliations:** 1 Center for Infectious and Inflammatory Diseases, Institute of Biosciences and Technology, Texas A&M Health Science Center, Houston, TX, 77030, United States of America; 2 Dep. of Molecular Medicine, UdR INSTM, Center for Tissue Engineering (C.I.T.), University of Pavia, 27100, Pavia, Italy; 3 Dep. of Occupational Medicine, Ergonomy and Disability, Salvatore Maugeri Foundation, IRCCS, Nanotechnology Laboratory, 27100, Pavia, Italy; 4 Department of Biology, University of York, York, YO10 5DD, United Kingdom; 5 Wellcome Trust Centre for Cell-Matrix Research, Faculty of Life Sciences, University of Manchester, Manchester, M13 9PT, United Kingdom; Institut Albert Bonniot-INSERMU823, FRANCE

## Abstract

Adherence of microbes to host tissues is a hallmark of infectious disease and is often mediated by a class of adhesins termed MSCRAMMs (Microbial Surface Components Recognizing Adhesive Matrix Molecules). Numerous pathogens express MSCRAMMs that specifically bind the heterodimeric human glycoprotein fibronectin (Fn). In addition to roles in adhesion, Fn-binding MSCRAMMs exploit physiological Fn functions. For example, several pathogens can invade host cells by a mechanism whereby MSCRAMM-bound Fn bridges interaction with α_5_β_1_ integrin. Here, we investigate two Fn-binding MSCRAMMs, FnBPA (*Staphylococcus aureus*) and BBK32 (*Borrelia burgdorferi*) to probe structure-activity relationships of MSCRAMM-induced Fn/α_5_β_1_integrin activation. Circular dichroism, fluorescence resonance energy transfer, and dynamic light scattering techniques uncover a conformational rearrangement of Fn involving domains distant from the MSCRAMM binding site. Surface plasmon resonance experiments demonstrate a significant enhancement of Fn/α_5_β_1_ integrin affinity in the presence of FnBPA or BBK32. Detailed kinetic analysis of these interactions reveal that this change in affinity can be attributed solely to an increase in the initial Fn/α_5_β_1_ on-rate and that this rate-enhancement is dependent on high-affinity Fn-binding by MSCRAMMs. These data implicate MSCRAMM-induced perturbation of specific intramolecular contacts within the Fn heterodimer resulting in activation by exposing previously cryptic α_5_β_1_ interaction motifs. By correlating structural changes in Fn to a direct measurement of increased Fn/α_5_β_1_ affinity, this work significantly advances our understanding of the structural basis for the modulation of integrin function by Fn-binding MSCRAMMs.

## Introduction

**Fig 1:**
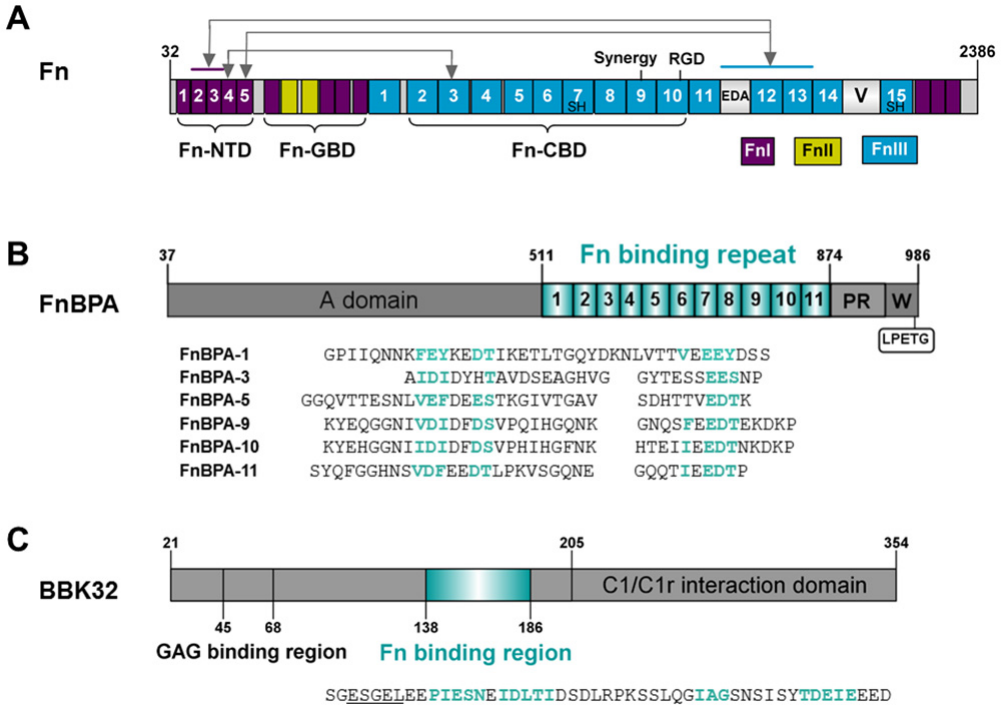
Domain architectures and sequences of the polypeptides. (**A**) Fn is a large C-terminally disulfide linked heterodimer that is modular in nature. Alternative splicing results in Fn monomers which are similar but not identical [5]. For clarity, only the larger monomer is shown here. Fn consists of three domain types each with distinct folds: FnI, FnII and FnIII (Uniprot ID: P02751). Splice variation results in a variable region (V) in a single Fn monomer. Several functionally relevant protease-stable Fn fragments are known, including the N-terminal domain (Fn-NTD), the gelatin-binding domain (Fn-GBD), and the cell-binding domain (Fn-CBD). The Fn-CBD fragment harbors the canonical α_5_β_1_ “RGD” interaction motif within the 10^th^ FnIII module and “synergy site” in the 9^th^ FnIII module. Two free cysteines (SH) exist in each Fn monomer which were used to site-specifically label the FRET probes used in this study and are located in the 7^th^ and 15^th^ FnIII modules. Arrows indicate specific intramolecular interactions formed between domains of the Fn-NTD fragment and the distant Fn-CBD fragment. (**B**) Domain organizations of mature FnBPA from *S*. *aureus*. The extracellular region of FnBPA consists of an A domain that interacts with fibrinogen, followed by a sequential arrangement of 11 Fn binding repeats, the proline-rich (PR) and cell wall (W) spanning regions, and a C-terminal sortase A recognition motif (LPETG) (Uniprot ID: P14738). Sequence alignment of Fn-binding repeats used in this study are shown. Highlighted in cyan are the residues within each repeat that bind to or are presumed to engage Fn-NTD. (**C**) Domain organizations of mature BBK32 from *B*. *burgdorferi* (Uniprot ID: O50835). The residues involved in binding to Fn-NTD are shown in cyan. Unlike FnBPA BBK32 also encodes a binding site for Fn-GBD indicated by the underlined sequence [6]. The recently identified C1 binding domain is also indicated [7]. All domains are drawn to the scale using IBS (Illustrator for Biological Sequences, http://ibs.biocuckoo.org).

Fibronectin (Fn) is a multidomain glycoprotein found in blood plasma, other bodily fluids and the extra-cellular matrix (ECM) and serves as a natural ligand for several integrins including α_5_β_1_, α_v_β_3_, and α_4_β_1_ [1]. The functional roles of Fn are diverse and include ECM assembly, angiogenesis, wound-repair, and oncogenesis [2]. Fn is secreted as a C-terminally disulfide-linked heterodimer composed of three structurally defined repeating units termed Fn domains or modules (Fig 1A). Major forms of Fn are comprised of 12 type I modules (FnI) and two type II modules (FnII), while splice variation results in 15 to 17 type III modules (FnIII). Fn is found in two predominant forms; cellular Fn, which is tissue localized and assembled into a fibrillar matrix, and the hepatocyte expressed, soluble plasma Fn that is secreted and maintained in blood at ~0.3 mg ml^-1^ [3]. Despite having independent functional roles from cellular Fn, it is significant that plasma Fn accounts for a large fraction of Fn found in tissue ECM [3,4].

To date, over one-hundred bacterial Fn-binding proteins (FnBPs) have been reported, and a majority of these belong to a protein family termed Microbial Surface Components Recognizing Adhesive Matrix Molecules (MSCRAMMs) [2]. Many Fn-binding MSCRAMMs contribute to virulence as *in vivo* studies demonstrate a critical role for these proteins in development of infectious diseases such as endocarditis, mastitis, and wound-infection [8–10]. A substantial body of evidence suggests that FnBPs can also manipulate physiological functions of Fn and thus contribute to pathogenesis in ways beyond mediating bacterial adhesion [6,11–16]. Indeed, several studies over the past decade involving the F1 and SfbI proteins from *Streptococcus pyogenes* [11,12,17], and more recently, BBK32 from *Borrelia burgdorferi* [6], have led to the development of a model whereby certain endogenous Fn activities are activated allosterically by Fn-binding MSCRAMMs. Specific intramolecular interactions exist in native plasma Fn which hold its solution conformation in a relatively compact state [18]. These interactions are mediated, in part, by FnI modules from the Fn-NTD fragment and FnIII modules originating from the distant Fn-CBD fragment (Fig 1A) [19–22]. Intrinsically disordered sequences from SfbI, F1, and BBK32 engage the Fn-NTD fragment via a tandem β-zipper model of binding [6,23–25] and, in doing so, compete for these intramolecular Fn contacts [6,12,24]. This competition results in a conformational expansion of Fn which has been directly measured using dynamic light scattering (DLS) upon binding of SfbI [12]. Interestingly, the SfbI-induced conformational change in Fn mirrors the transition of compact Fn to an elongated structure in solutions of increasing ionic strength [18].

A monoclonal antibody (mAbIII-10), which recognizes a conformationally-sensitive epitope within ^10^FnIII [26] has also been used to monitor structural changes induced in Fn by both streptococcal and borrelial FnBPs [6,12,17]. These studies reveal a conformational rearrangement in Fn that occurs in domains located far outside of the FnBP/Fn binding site. The conformational expansion of Fn induced by binding of SfbI, F1, or BBK32 has been linked to three primary effects; (i) the 10^th^ FnIII module of Fn which harbors the “RGD” integrin recognition motif exposes a previously cryptic mAbIII-10 epitope, (ii) the motogenic “IGD” motifs of the 7^th^ and 9^th^ FnI modules of the Fn-GBD fragment become exposed, and (iii) in the case of SfbI, binding results in the blocking of Fn assembly into fibrils. In addition to binding to the Fn-NTD fragment, SfbI, F1 and BBK32 also harbor an Fn-GBD binding site. However, Fn-NTD interaction alone is sufficient to induce allosteric changes in Fn [12]. Consistent with this is the recent discovery of the *Streptococcus equi* FnBP termed SFS, which only binds Fn via Fn-GBD interaction, yet fails to cause conformational expansion of Fn [27].

Integrins are essential metazoan heterodimeric glycoproteins that mediate cell-adhesion, establish transmembrane connections to the cytoskeleton, and play an integral role in cell signaling pathways [28]. Interestingly, integrins are common targets of pathogens and often participate in bacterial and viral adhesion to host cells [29]. Two modes of microbial integrin recognition have been described and include direct binding by bacterial surface proteins [30–32] or indirect binding via physiological ligands such as Fn [29,33,34]. The latter mode is exemplified by an FnBP expressed by the gram-positive pathogen *Staphylococcus aureus*, termed FnBPA [35]. By acting as a molecular bridge, staphylococcal FnBPA exploits Fn’s role as a natural substrate for α_5_β_1_ integrin, causing activation of endocytic pathways, and ultimately resulting in the internalization of *S*. *aureus* by non-professional phagocytes [14–16,36–45]. Full-length FnBPA harbors eleven Fn-binding repeats that specifically interact with the ^2-5^FnI modules of the Fn-NTD fragment via a tandem beta-zipper [25,46] (Fig 1B). Unlike the streptococcal and borrelial FnBPs, FnBPA binds exclusively to Fn-NTD and makes no direct interactions with the Fn-GBD fragment. Of the 11 FnBPA Fn-binding repeats, six exhibit high-affinity binding of Fn [46] and a single high-affinity repeat is sufficient for α_5_β_1_ integrin-dependent *in vitro* invasion of endothelial cells by *S*. *aureus* [14].

While *S*. *aureus* is typically considered an extracellular pathogen, intracellular *S*. *aureus* is associated with several chronic and reoccurring infections [47–50] and can establish infection even in the presence of the vancomycin [51]. Thus, *S*. *aureus* internalized by non-professional phagocytes represents a bacterial reservoir protected against antibiotics and innate host defense systems. Although it is evident that FnBPA, Fn, and α_5_β_1_ integrin work in concert to promote an intracellular “life-style” for *S*. *aureus* [14–16,40], detailed knowledge of the underlying molecular events that lead to invasion have remained elusive. A mechanistic understanding of how FnBPA manipulates Fn function to enable entry of *S*. *aureus* into non-phagocytic cell types, will benefit renewed efforts [51] to target this form of the pathogen. With this in mind, the goals of this study were to determine if the allosteric model of Fn activation, which has been proposed for streptococcal and borrelial FnBPs, applies to staphylococcal FnBPA. Furthermore, we sought to better understand the nature of structural changes induced by Fn-binding MSCRAMMs and correlate these changes to quantitative and direct measurements of Fn/α_5_β_1_ integrin interaction. Therefore, in parallel with our studies of FnBPA, we have also evaluated the effect of borrelial BBK32 (Fig 1C) on the solution structure of Fn and its effect on Fn/α_5_β_1_ interaction. Herein, we apply a cross-disciplinary approach to dissect the structural basis for MSCRAMM-induced Fn activation using circular dichroism (CD), DLS, and fluorescence resonance energy transfer (FRET) to examine the conformational status of native Fn or MSCRAMM-bound Fn in solution. Surface plasmon resonance (SPR) is used to quantitatively measure the effects of FnBPA and BBK32 on direct Fn/α_5_β_1_ interaction. Together these experiments provide a detailed structure-activity relationship of MSCRAMM/host interaction and strongly suggest an allosteric mode of Fn/α_5_β_1_ affinity-enhancement for Fn-binding MSCRAMMs.

## Materials and Methods

### Materials

Human Fn was purified from freshly drawn citrated plasma (Gulf Coast Regional Blood Center, Houston, TX) using gelatin affinity chromatography combined with arginine affinity chromatography as described previously [52]. Fn was stored at 4°C in Tris-buffered saline (TBS: 50 mM Tris-HCl, pH 7.4, 150 mM NaCl), and used within one month. Fn dimer concentration was calculated from OD_280_ nm with EC_280_ (1%) = 12.8 and molecular weight of 500 kDa. The ecto-domain of integrin α_5_β_1_-Fc fusion protein, which contains the N-terminal 613 residues of α_5_-subunit and fragment 121–455 of β_1_-subunit, was generated and purified as previously reported [53]. Fn-NTD and Fn-CBD were purified as previously described [54]. Expression and purification of recombinant GST-FnBPAs, FnBPA-10 peptide and BBK32_(21–205)_ was performed as previously described [46,55]. Rabbit anti-Fn pAb (polyclonal antibody) was purchased from ICN Pharmaceuticals, Inc. (Costa Mesa, California). BSA (bovine serum albumin), heparin sodium (from porcine intestinal mucosa, 17,000–19,000 Da) was from Sigma.

### Dynamic light scattering

DLS measurements were performed using DynaPro Titan Ambient laser unit (Wyatt Technology Corporation) with wavelength at 828.5 nm and 10% to 12% laser power. Samples were dissolved in TBS and filtered through 0.22 μm filter to eliminate any particles. Fn concentration was maintained at 0.8 mg ml^-1^ (1.6 μM as dimer) in TBS (50 mM Tris-HCl pH 7.4, 150 mM NaCl) for the conditions tested. After equilibration at room temperature for 15 to 30 min, the samples were measured three times, with each measurement lasting 100 seconds (10 acquisitions, 10 seconds each). Data analysis was performed using Dynamics V6 software.

### Circular Dichroism

CD measurements in the near UV region (350–250 nm) were carried out at ambient temperature on a Jasco J-720 spectropolarimeter (Easton, MD) with a 1 cm cell. Ten scans were collected and averaged at a scan speed of 200 nm min^-1^, with a time constant of 1 s and band width of 1 nm. A mean residue molecular weight of 110 was used in the calculation of mean residue ellipticity. The spectra of the Fn solution were background-corrected with the CD signal obtained from the buffer or molecules mixed with Fn.

### Fluorescence resonance energy transfer

Fluorescence labeling of human plasma Fn was performed at room temperature as described previously [56]. The fluorescence probes used in this study were purchased from Molecular Probes. Briefly, newly purified human plasma Fn at 1.2 mg ml^-1^ in TBS was partly denatured in 4 M GdnHCl (Sigma) for 15 min to expose the free cysteines in the Fn dimer. The denatured Fn was mixed with thiol-reactive Alexa Fluo^®^ 546 C5-maleimide (AF546, ~15-fold molar excess over Fn monomer concentration) and allowed to incubate for 2 hr with gentle rocking. The acceptor fluorophore AF546-conjugated Fn (~0.9 mg ml^-1^) was labeled with a second probe, the amine-reactive Alexa Fluo^®^ 488 carboxylic acid-succinimidyl ester-mixed-isomers (AF488, ~20-fold molar excess). After each labeling, the unbound dye was removed using a PD-10 desalting column (GE Healthcare). The labeling ratios were ~8 AF488 donors and ~4 AF546 acceptors in each Fn dimer.

FRET within donor and acceptor-labeled Fn was measured using spectrofluorimeter LS 50B (Perkin-Elmer) at room temperature with excitation wavelength at 493 nm. The sensitivity of FRET response to the Fn unfolding was evaluated under mild denaturant (up to 2 M guanidinium chloride (GdnHCl)). Measurement of MSCRAMM-induced FRET within Fn was performed by titrating 0.5, 2 and 5 μl of 50 μM of BBK32 or FnBPA-10 in to 500 μl of labeled Fn. Subsequently, the emission spectra collected for each titration was recorded and normalized to the donor emission peak so that changes in energy transfer were reflected only by changes in the acceptor peak. Control experiments were conducted using an equimolar solution of donor-labeled Fn with acceptor-labeled Fn in the presence and absence of 2M GdnHCl to confirm the absence of energy transfer between adjacent Fn molecules in solution.

### SPR-based Biacore analysis of Fn and α_5_β_1_ interaction

Experiments were performed at 25°C on a Biacore 3000 (GE Healthcare Bio-Sciences AB, Uppsala, Sweden) using HBS-T (10 mM HEPES, pH 7.3, 150 mM NaCl, 0.005% Tween-20) containing 1 mM MnCl_2_. To prepare sensor surface for kinetic experiments, ~1400 RU of α_5_β_1_ was immobilized to a CM5 chip by amine coupling. Briefly, 18 μL of protein (10 μg ml^-1^ in 10 mM sodium acetate, pH 4.8) was injected onto an activated (4 min activation) surface at a flow rate of 5 μl min^-1^. BSA was coupled to the adjacent flow cell and served as a reference surface (~5000 RU). A flow rate of 30 μL min^-1^ was used for all binding experiments. The sensor chip surface was regenerated to remove bound Fn by injection of 1 M NaCl for 1 min. All measurements were baseline corrected by subtracting the response from the reference surface. For kinetic analysis, signals from buffer blanks were subtracted.

Kinetic constants were obtained from curve fitting to the predefined bivalent analyte model using BIAevaluation software (Version 4.1). Heterodimeric Fn has two identical binding sites for integrin α_5_β_1_. The reaction between soluble Fn analyte (A) and immobilized integrin ligand (L) can be described by this equation:

$$\text{A}+\text{L }\overset{k_{a1}}{\underset{k_{d1}}{↔}}\text{ AL}+\text{L }\overset{k_{a2}}{\underset{k_{d2}}{↔}}\;{\text{AL}}_{2}$$

Binding to the first ligand molecule is described by a single set of rate constants (*k*_a1_, *k*_d1_), so that the two sites on the analyte are equivalent in the first binding step. Binding of the second ligand molecule is described by a second set of rate constants (*k*_a2_, *k*_d2_), which is reported in an unconventional units of RU^-1^s^-1^. The first set of rate constants were used for calculating the dissociation constant *K*_D_ (*K*_D_ = *k*_d1_/*k*_a1_).

### ELISA-type binding assays

Experiments were conducted at room temperature and each sample was assayed in triplicate. Immunoassay microplate Immulon 2HB (Thermo Scientific, Waltham, MA) was coated with 2 μg of integrins dissolved in TBS for 1 hour. The plate was then blocked for 1 h with 1% (w/v) BSA in a buffer “A” (TBS containing 1 mM MnCl_2_). Citrated plasma (stored at -20°C for less than 6 months) was fast-thawed at 37°C and spun to remove aggregates. The supernatant was diluted 10-fold in buffer “A” and then mixed with varying concentrations of BBK32 to a final concentration of 5% plasma. The sample mixtures were added onto the plate and incubated for 1 h. The plate was washed three times with buffer “B” (TBS with 1 mM, MnCl_2_, and 0.05% Tween-20) before addition of rabbit anti-Fn antibody. Following washing with buffer “B”, AP-conjugated goat-anti-rabbit IgG (BioRad, Hercules, CA) was added to the plate and incubated for 1 h. All antibodies were diluted 2000-fold in buffer “B” containing 1% BSA. Alkaline phosphatase substrate PNPP (Pierce, Rockford, IL) was added after washing, and the absorbance was measured at 405 nm using a Thermo Max plate reader (Molecular Devices, Sunnyvale, CA).

## Results

### The tertiary structure of plasma Fn is altered by FnBPA and BBK32

Although high resolution crystal structures have been solved for a number of smaller Fn fragments, a complete structure of full-length Fn remains to be determined. However, a wide range of biophysical studies using DLS [18,57–61], CD [59,62,63], steady-state fluorescence spectroscopy [59,63,64], small-angle X-ray scattering (SAXS) [65] and small-angle neutron scattering (SANS) [60,65,66] have yielded a wealth of information about the conformational states and structure of native Fn in solution. Conformational changes induced by FnBPs underlie the allosteric model of Fn activation proposed for streptococcal and borrelial FnBPs [6,12,17], and a previous study using DLS indicates an expansion of Fn structure upon SfbI binding which is similar to what is observed when Fn is incubated in a high salt medium [12]. To understand if analogous conformational changes are induced by staphylococcal FnBPA we used DLS to measure the hydrodynamic radii (*R*_h_) of Fn in the presence or absence of the FnBPA Fn-binding repeat FnBPA-10 (Table 1). Fn adopted an extended conformation in the presence of high salt or heparin (*R*_h_ = 13.27 ± 0.15; 12.50 ± 0.29, respectively) compared to a more compact structure found in physiological buffer (*R*_h_ = 10.20 ± 0.12) which is consistent with previous measurements of Fn *R*_h_ by DLS [12,18]. Addition of staphylococcal FnBPA-10 (*R*_h_ = 12.73 ± 0.69) or the borrelial FnBP, BBK32 (*R*_h_ = 13.00 ± 0.38) resulted in a significantly increased *R*_h_, suggesting that Fn indeed adopts an extended structure upon binding to these MSCRAMMs.

**Table 1:** Comparison of hydrodynamic radii of Fn under various conditions.

Fn treatments	*R*_h_ (nm)
TBS	10.20 ± 0.12
High salt	13.27 ± 0.15
Heparin	12.50 ± 0.29
FnBPA-10	12.73 ± 0.69
BBK32	13.00 ± 0.38

Dynamic light scattering measurements were performed using DynaPro Titan Ambient laser unit. Fn was maintained in TBS at a final concentration of 0.8 mg ml^-1^ (1.6 μM) for the conditions tested. Different treatments are additional 350 mM NaCl (high salt), 15 μM of heparin, FnBPA-10 peptide, or 6 μM of BBK32 recombinant protein. Values are mean ± SE obtained from a minimum of three independent experiments.

**Fig 2:**
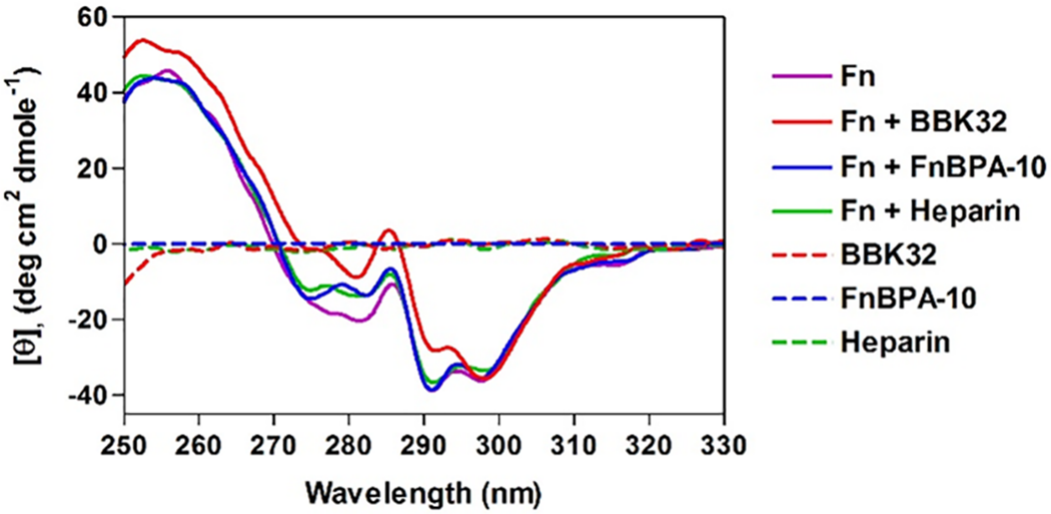
Near-UV CD spectra of Fn. CD spectra in the range of 250–350 nm are shown for individual molecules or mixtures: Fn dimer (0.82 mg ml^-1^, 1.6 μM), BBK32 (0.14 mg ml^-1^, 6.0 μM), FnBPA-10 (0.07 mg ml^-1^, 15 μM), and heparin (0.27 mg ml^-1^, 15 μM). All concentrations are final. TBS (50 mM Tris-HCl pH 7.4, 150 mM NaCl) was used for all experiments.

Native Fn contains large numbers of aromatic residues in its type I and III domains which exist in common hydrophobic environments and give rise to a signature near-UV CD spectrum characterized by two strong negative peaks centered at 291 nm and 299 nm, and a weaker negative peak at 282 nm (Fig 2) [67,68]. Binding of Fn by the glycosaminoglycan heparin has been shown to alter Fn tertiary structure which can be monitored by shifts in this signature spectrum (Fig 2) [69]. When Fn was incubated in the presence of FnBPA-10 or BBK32, both complexes produce significantly different spectra relative to native Fn (Fig 2). Interestingly we noted the similarity in the near-UV spectra for heparin-bound Fn when compared to FnBPA-10/Fn (Fig 2), whereas, the BBK32 spectrum is reminiscent of the partial unfolding of Fn in 4M urea [63]. Importantly, the MSCRAMM fragments used in these experiments are intrinsically disordered polypeptides that do not contain tryptophan residues and therefore lack near-UV optical activity [70] (Fig 2). However, since the Fn-NTD is engaged by BBK32 and FnBPA-10, such interaction could potentially cause near-UV CD optical activity changes within NTD domain. As shown in S1 Fig, BBK32 and FnBPA-10 caused intrinsic Trp fluorescence quenching in Fn (S1A Fig) and Fn-NTD (S1B Fig), suggesting that Trp environments in the NTD and GBD (in the case for BBK32) are affected by the binding and may attribute partially to the near-UV CD changes in Fn.

**Fig 3:**
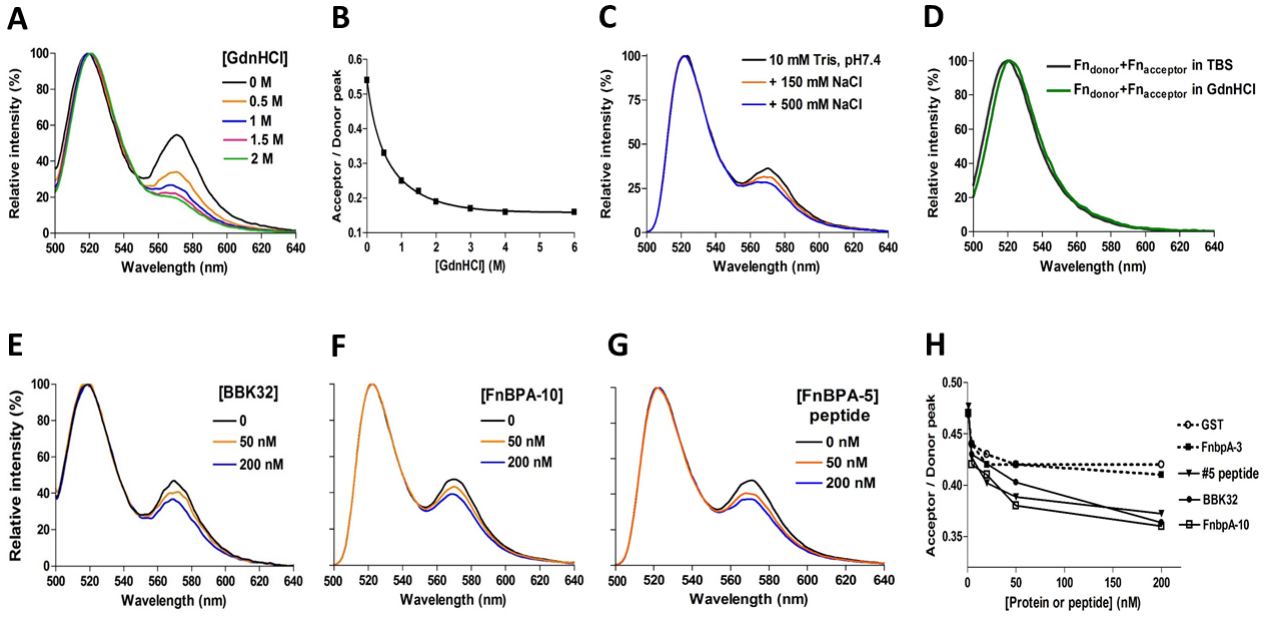
Fluorescent resonance energy transfer of Fn. Fluorescence emission spectra of Fn-D/A (50 nM in 10 mM Tris, pH7.4, 150 mM NaCl) were collected at 493 nm excitation, (A) in the presence or absence of denaturant (GdnHCl) and (B) accompanying Acceptor/Donor peak ratio as a function of denaturant concentration. Fn-D/A fluorescence emission changes (C) under different ionic strengths, and (E-G) in the presence of different concentrations of BBK32, GST-FnBPA-10, or FnBPA-5 peptide. (H) Acceptor/Donor peak ratio vs. concentration (0, 1, 5, 20, 50 and 200 nM) of protein or peptide added. Spectra are normalized to the donor emission peak at 522 nm such that changes in energy transfer are reflected only by changes in the acceptor peak at 570 nm. (D) Control experiments were conducted by mixing 50 nM of donor-labeled Fn (Fn_donor_) with 50 nM of acceptor-labeled Fn (Fn_acceptor_) in TBS or 2 M GdnHCl.

Data obtained using an anti-Fn antibody (mAb10-III) that recognizes a conformational sensitive epitope indicates that structural changes distant from the streptococcal and borrelial FnBP binding site are induced in Fn [6,12,17] on MSCRAMM binding. To learn if FnBPA causes similar allosteric changes in Fn upon binding, and to confirm apparent conformational changes in solution Fn indicated by CD (Fig 2), we next employed a FRET-based approach. To this end, Fn was site-specifically labelled by conjugating an acceptor fluorophore to free cysteine residues using a technique previously described [56]. Native Fn contains only two free cysteines (4 per dimer molecule) and these are found in the C-terminally positioned ^7^FnIII and ^15^FnIII modules [71] (denoted in Fig 1A). When Fn is denatured by introducing increasing amounts of GdnHCl (Fig 3A and 3B), or incubated in the presence of increasing ionic strength (Fig 3C), the distance between the amine coupled donor fluorophores and the cysteine coupled acceptor fluorophores increased and the acceptor peak was diminished. There is not FRET observed between adjacent or denatured Fn molecules (Fig 3D). To determine if the MSCRAMM-induced structural changes measured by CD and DLS involve Fn domains outside of the MSCRAMM binding site, spectra were then collected in the presence of various concentrations of BBK32 (Fig 3E) and FnBPA-10 (Fig 3F) GST fusion proteins. These experiments showed a clear dose-dependent loss of acceptor peak signal for MSCRAMM-bound Fn. Importantly, an identical effect is observed when a 36 amino acid peptide lacking the GST fusion and corresponding to the high-affinity FnBPA repeat FnBPA-5 is used (Fig 3G). GST alone or a GST fusion to the low-affinity FnBPA repeat FnBPA-3 exhibits no effect in this assay system (Fig 3H). Taken together, the DLS, CD, and fluorescence based assays indicated that BBK32 and FnBPA profoundly modify the native solution structure of Fn by promoting an extended Fn conformation that results in large scale rearrangement of Fn domains at sites distant from the MSCRAMM binding site.

### Fn recognition of the α_5_β_1_ integrin is enhanced by FnBPA and BBK32

Fn serves as a primary ECM substrate for several adhesion molecules including the major Fn cell surface receptor, α_5_β_1_ integrin [1]. We hypothesized that conformational changes induced upon binding by FnBPA or BBK32, as shown in Figs 2 and 3 and Table 1, may result in modified Fn/integrin interactions. Previous studies on streptococcal and borrelial Fn-binding MSCRAMMs have used the conformational Fn antibody (mAb10-III) as a surrogate for potential effects on Fn/α_5_β_1_ integrin interaction. To test this more directly, we developed an SPR-based system to study the effects of FnBPA and BBK32 on the recognition of α_5_β_1_ integrin by Fn. Briefly, a recombinant α_5_β_1_-Fc fusion protein representing the minimal functional unit of the integrin [53] was immobilized on the surface of a Biacore CM5 chip. Fn interacts with the α_5_β_1_ biosensor and binding is enhanced by Mn^2+^ as previously reported (S1A Fig) [53]. Further validation of this experimental approach was performed by using Fn fragments including the cell-binding domain (Fn-CBD) possessing the canonical α_5_β_1_-binding “RGD” (^10^FnIII) and PSHRN synergy sites (^9^FnIII) [72,73]. As expected, the Fn-CBD fragment exhibited dose-dependent interaction with α_5_β_1_, while the Fn-NTD fragment lacking the integrin interaction domains failed to bind the integrin (S1B and S1C Fig).

**Fig 4:**
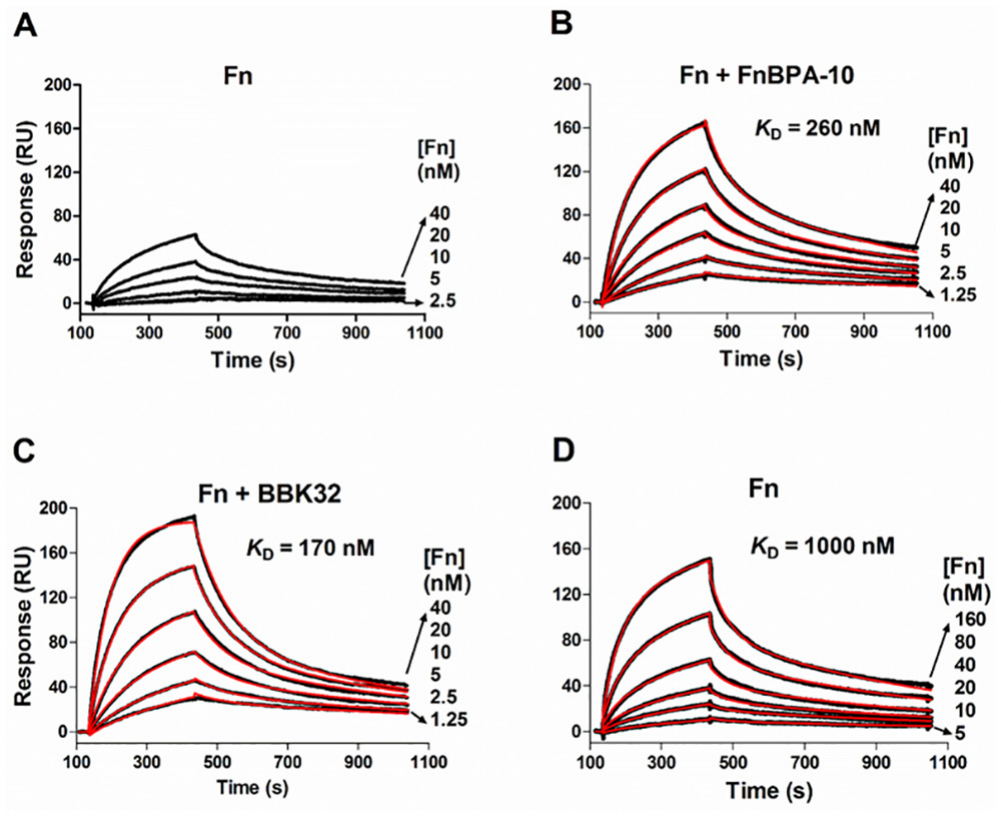
SPR analysis of direct Fn/α_5_β_1_ interaction in the presence of Fn-binding MSCRAMMs. **(A)** Two-fold linear dilution series of Fn in HBS-T containing 1 mM MnCl_2_, and in the presence of **(B)** 1 μM of FnBPA-10, or **(C)** 0.5 μM of BBK32 were injected over the α_5_β_1_ biosensor. **(D)** A higher Fn concentration series was used for measurement of Fn/α_5_β_1_ binding kinetics in the absence of MSCRAMMs. The SPR response curves generated from Fn binding to α_5_β_1_ are shown in black with lower curve corresponding to lower concentration of Fn injected. Dissociation constants (*K*_D_) for Fn/α_5_β_1_ the interaction **(B-D)** were obtained from fitting SPR response curves to a bivalent analyte binding model (fitted lines are shown in red) and the kinetic parameters are listed in Table 2. There was no detectable binding when FnBPA-10 (1 μM) or BBK32 (0.5 μM) were injected over the same α_5_β_1_ surface (data not shown).

Using this approach we measured a moderate affinity of ~1 μM for Fn/α_5_β_1_ (Fig 4A–4D) that is in good agreement with affinities reported for fibroblast cell interaction with plasma Fn [74]. To understand if MSCRAMM binding to Fn affects the Fn/α_5_β_1_ interaction, we next injected mixtures of BBK32/Fn or FnBPA-10/Fn (fixed MSCRAMM + varied Fn concentrations) over the immobilized α_5_β_1_ integrin. A remarkable enhancement of the binding response was observed for MSCRAMM-bound Fn relative to native Fn (Fig 4B–4D). To obtain a quantitative measure of the increase in affinity, kinetic parameters were obtained by fitting SPR response curves to a bivalent analyte binding model (Fig 4B–4D). This model was selected on the basis of closeness of fit (*χ*^2^≈1, Table 1) and the expectation that dimeric Fn contributes one identical α_5_β_1_ binding site per subunit. Analysis for each interaction revealed that the approximately five-fold increase in apparent affinity (*K*_D_) (*K*_D_^Fn/α5β1^ = 1000 nM vs. *K*_D_^BBK32/Fn/α5β1^ = 170 nM & *K*_D_^FnBPA-10/Fn/α5β1^ = 260 nM) was due to an increase in the initial association rate ([*k*_a1_ = (1.5, 6.1, & 7.5) x10^4^ M^-1^s^-1^], respectively). Interestingly, all other rate constants (*k*_d1_, *k*_a2_ and *k*_d2_) (Table 2) were similar across experiments, indicating that the Fn/α_5_β_1_ binding site present in native Fn is the same for MSCRAMM-bound Fn.

**Table 2:** Kinetic parameters for Fn::α_5_β_1_ interactions.

Analyte injected	*k*_a1_(×10^4^ M^-1^s^-1^)	*k*_d1_ (×10^−2^ s^-1^)	*k*_a2_ (×10^−5^ RU^-1^s^-1^)	*k*_d2_ (×10^−3^ s^-1^)	*K*_D_ (nM)	χ ^2^
**Fn alone**	1.5 ± 0.0	1.5 ± 0.0	1.1 ± 0.1	0.9 ± 0.0	1000 ± 130	1.15 ± 0.26
**Fn + FnBPA-10**	6.1 ± 0.6	1.5 ± 0.2	1.3 ± 0.3	1.0 ± 0.1	260 ± 90	1.10 ± 0.29
**Fn + BBK32**	7.5 ± 0.8	1.2 ± 0.2	0.8 ± 0.0	1.0 ± 0.1	170 ± 90	1.04 ± 0.42

Association (*k*_a1_, *k*_a2_) and dissociation rate (*k*_d1_, *k*_d2_) constants obtained from fitting SPR response curves (Fig 4B–4D) to a bivalent analyte binding model. The statistical value χ^2^ is also listed to show the closeness of each fit. Values are mean values (with standard error in parentheses) obtained from at least three experiments.

**Fig 5:**
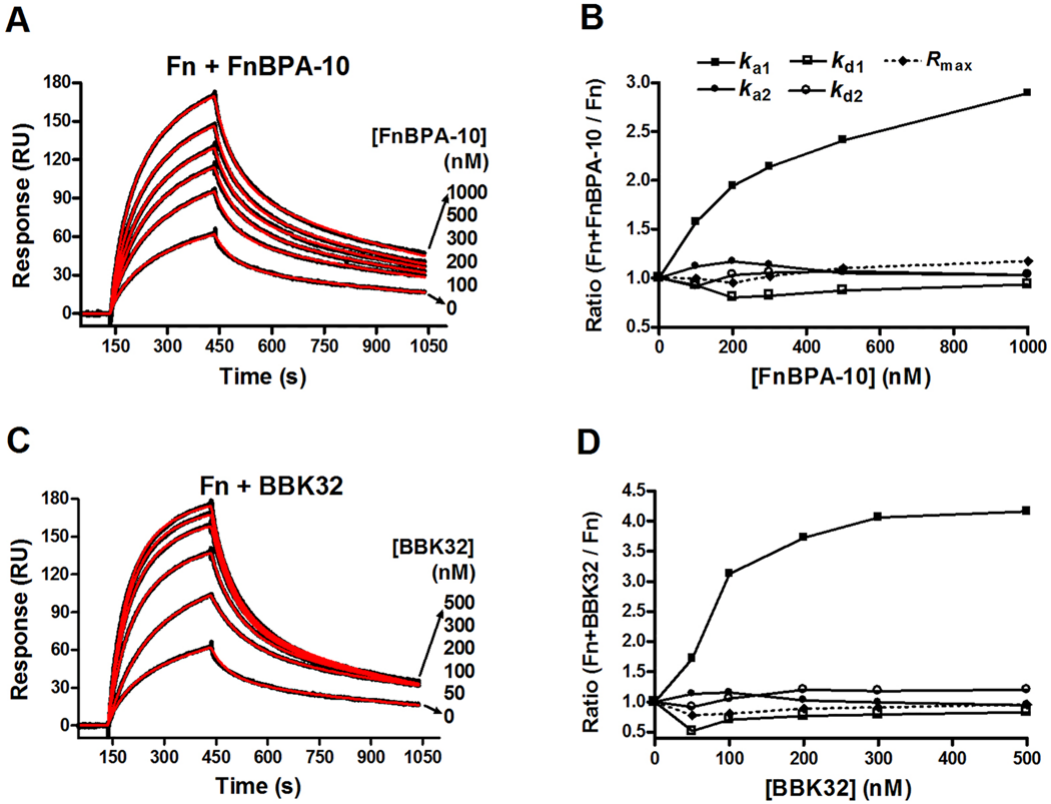
SPR analysis of kinetic components of the Fn/α_5_β_1_ interaction that are affected by MSCRAMMs. Fn (50 nM) in the presence of indicated concentration of **(A)** FnBPA-10 or **(C)** BBK32 was injected over an α_5_β_1_ surface on a Biacore sensor chip. Kinetic components (*k*_a1_, *k*_d1_, *k*_a2_, *k*_d2_) and R_max_ obtained from local fitting were normalized to those of Fn alone, and plotted as a function of **(B)** FnBPA-10 or **(D)** BBK32 concentrations.

To address the specificity of enhanced Fn/α_5_β_1_ activity and to further dissect the kinetic observations presented above, we next injected a mixture of varied MSCRAMM and fixed Fn concentrations (Fig 5A–5C). Again we observed an increase in response that behaved in a dose-dependent and saturable manner, similar to the results obtained in Fig 4B–4D. Likewise, only the initial association rate *k*_a1_ was changed, while the stability of the complex (*k*_d1_), the second rate step (*k*_a2_ and *k*_d2_), and maximum response when all the binding sites on active α_5_β_1_ are saturated (*R*_max_) were unaffected by the presence of MSCRAMMs (Fig 5B–5D). The initial association rate, *k*_a1_, is the only kinetic parameter directly affected by analyte concentration (units = M^-1^s^-1^). As the concentrations of Fn used across experiments were the same, the observed increase in *k*_a1_ for Fn bound by increasing concentrations of MSCRAMMs likely represents an increase in the apparent concentration of the active integrin binding site in Fn.

### High-affinity FnBPA/Fn-NTD interaction is required for enhancement of Fn/α_5_β_1_ activity

**Fig 6:**
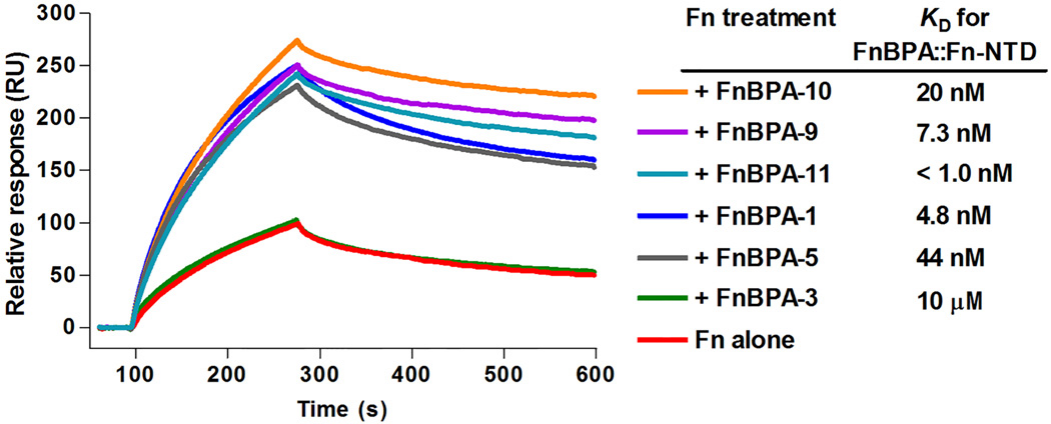
Relationship of high affinity Fn-NTD fragment interaction and α_5_β_1_ integrin activation. SPR sensorgrams were generated by injecting 50 nM Fn in the presence of GST-fused FnBPA repeats (0.5 μM for high-affinity repeats -1, -5, -9, -10, -11 and 15 μM for low affinity repeat -3) over immobilized α_5_β_1_. The *K*_D_ reported for direct binding of Fn-NTD by high affinity FnBPA repeats are provided for reference and were obtained from previously published isothermal titration calorimetry (ITC) experiments [46]. The weak affinity between FnBPA-3 and Fn-NTD was measured by SPR (this work, data not shown). None of the repeats bound directly to α_5_β_1_ (data not shown).

Of the eleven Fn-binding repeats found in FnBPA, six have been characterized as high-affinity binders (FnBPA-1, -4, -5, -9, -10, -11). The higher affinity of these repeats has been attributed to their ability to interact with four sequential FnI modules of the Fn-NTD fragment [46]. For example, while FnBPA-10 contains correctly spaced motifs to interact with ^2-5^FnI in a β-tandem zipper model of binding, the low-affinity repeat FnBPA-3 appears to lack the ^5^FnI interaction motif. Intriguingly, these particular Fn modules have been implicated in mediating long-range intra-molecular contacts within native Fn dimers (Fig 1A) [19,21,22]. To understand if high-affinity interaction is required for an MSCRAMM-induced increase in Fn*/*α_5_β_1_ recognition, we next tested a panel of high and low-affinity FnBPA repeats (Fig 6). All high-affinity repeats studied (FnBPA-1, -5, -9, -10, and -11) increased the Fn*/*α_5_β_1_ binding response, while the low-affinity repeat FnBPA-3 exhibited control-level signal.

### MSCRAMMs enhance Fn/α_5_β_1_ interaction in human plasma

**Fig 7:**
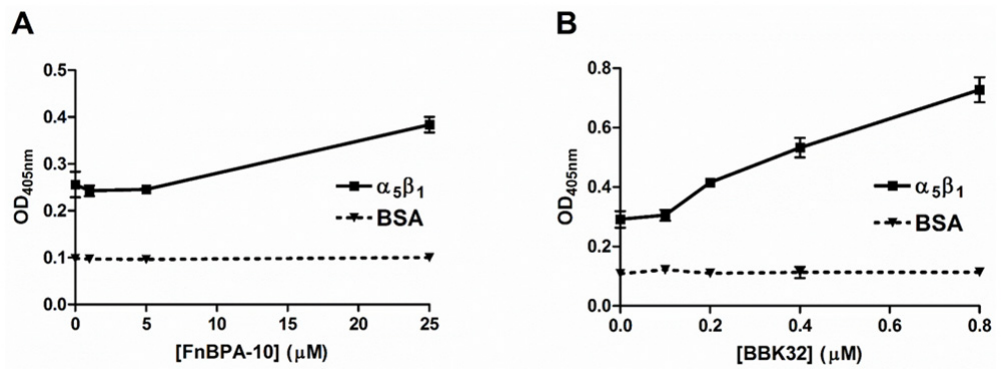
ELISA detection of the effect of MSCRAMMs on intact Fn in plasma for α_5_β_1_ binding. 5% human blood plasma in TBS containing 1 mM MnCl_2_ was incubated with **(A)** FnBPA-10 or **(B)** BBK32, and added α_5_β_1_-coated wells or BSA (0.2 μg per well) coated surfaces. Bound Fn was probed with anti-Fn pAb. BBK32 did not bind directly to α_5_β_1_ when detected by anti-BBK32 pAb (data not shown). Data are one representative of three experiments.

The SPR-based activity assay utilizing purified native and recombinant reagents allowed for a quantitative kinetic assessment of the MSCRAMM-induced enhancement of Fn/α_5_β_1_ binding. We next asked if this effect could be recapitulated using human plasma as a source of native Fn. Indeed, an MSCRAMM-dependent increase in plasma Fn/α_5_β_1_ binding was observed in the presence of FnBPA-10 or and to a greater extent for BBK32 when 5% human plasma (~15 μg ml^-1^ or equivalently ~30 nM Fn) was used in an ELISA-type binding assay (Fig 7).

## Discussion

### Modeling the allosteric activation of Fn/α_5_β_1_ by Fn-binding MSCRAMMs

**Fig 8:**
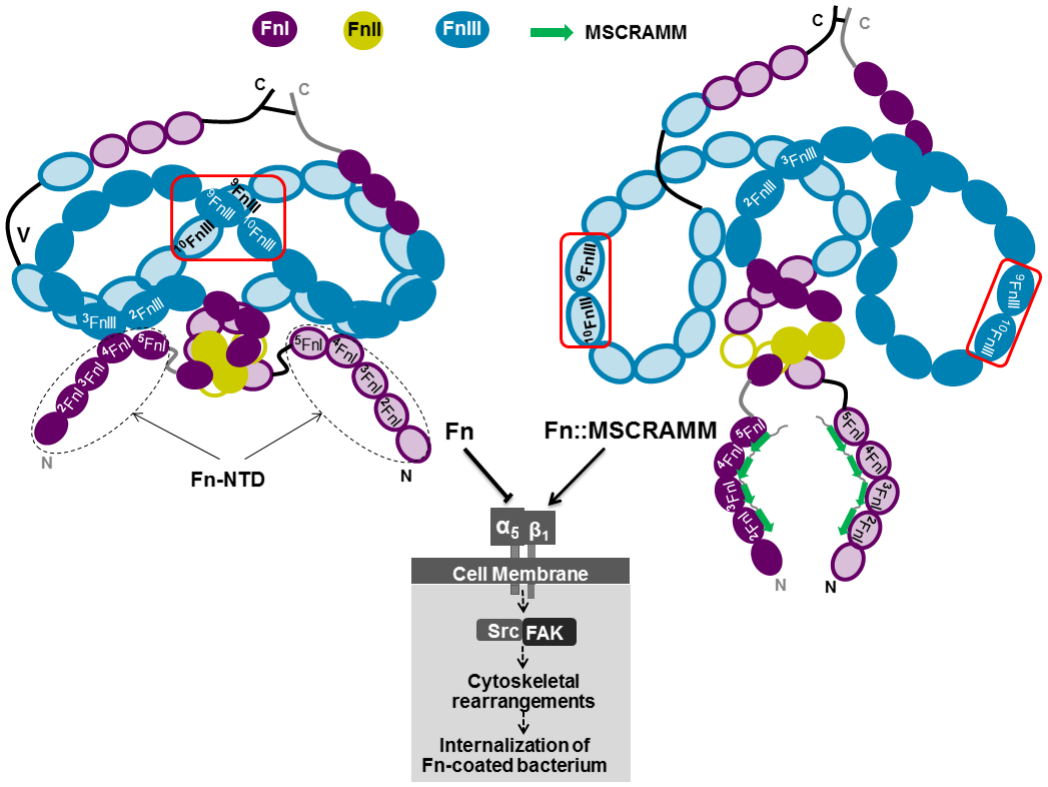
An allosteric model of Fn activation by MSCRAMMs. Based on the data presented here and the previously proposed allosteric model of Fn activation by streptococcal and borrelial FnBPs [6,12,17], we constructed the above model for allosteric affinity enhancement of the Fn/α_5_β_1_ interaction by staphylococcal FnBPA. Fn is represented in its native state (left) or bound by FnBPA (right). The native Fn model including intermolecular contacts are adapted from a previously proposed model based on multiple independent studies. In a native state, major solution conformations of Fn occlude the canonical α_5_β_1_ interaction motifs located in ^9-10^FnIII domains. Upon binding to Fn, FnBPA disrupts specific intermolecular contacts in the N-terminal domain of Fn (Fn-NTD) resulting in global structural rearrangements at sites distant from the FnBPA binding site. Previously cryptic integrin interaction sites are exposed, promoting interaction of Fn with α_5_β_1_. This initiates ‘outside-in’ signaling events leading to cytoskeletal rearrangement and eventual internalization of Fn-coated *S*. *aureus*.

By manipulating the conformation of key Fn functional domains, Fn-binding MSCRAMMs enable pathogens to hijack normal host physiology. In the case of *S*. *aureus*, enhanced Fn/α_5_β_1_ interaction leads to recruitment of focal contact-associated proteins and subsequent integrin clustering at the bacterial attachment site initiates intracellular signaling through the focal adhesion kinase (FAK) and Src kinases [33,43,75]. This appears to confer an advantage to *S*. *aureus* by facilitating immune evasion and serves as a bacterial reservoir in chronic infections [33,51]. In light of the observations above, we propose a model for the allosteric enhancement of Fn/α_5_β_1_ binding by staphylococcal FnBPA (Fig 8), similar to that put forward for streptococcal and borrelial FnBPs [6,12,13,17]. We speculate that dimeric native Fn exists as an array of conformers in solution. At equilibrium, predominate forms of Fn are compact and sterically occlude α_5_β_1_ interaction domains (^9^FnIII-^10^FnIII) by long-range intramolecular interactions, thereby impeding Fn/α_5_β_1_ recognition. The equilibrium of Fn conformers shifts upon high-affinity binding of FnBPA and native intramolecular contacts are disrupted exposing previously cryptic α_5_β_1_ binding sites and promoting Fn/α_5_β_1_ binding. Domains involved in integrin binding by Fn are located at a site distant from the MSCRAMM binding site. It appears then that a critical feature of MSCRAMM-induced activation of Fn/α_5_β_1_ is allostery and allosteric modes of host protein modulation have been previously reported for *S*. *aureus* [76]. Our findings also suggest that the conformation of integrin ligands, like Fn, may fine-tune affinities and therefore contribute to integrin “activation” traditionally associated with conformational change of the integrin ectodomains [77].

The data presented here provides a foundation to study the potential involvement of Fn-binding MSCRAMMs in several aspects of Fn physiology. In addition to Fn/integrin interaction, cryptic functional sites in Fn play a fundamental role in mechanoresponsive and motogenic pathways [19,78]. Interestingly, polymorphisms have been recently reported in two high-affinity FnBPA repeats (FnBPA-5 and -9) and *S*. *aureus* strains harboring these mutations are highly correlated with infection of cardiovascular devices [79,80]. How these FnBPA mutations impact bound Fn tertiary structure is currently unknown, however, we predict that sequence changes to residues in FnBPA repeats could result in different global Fn structure and thus function. Finally, as the clinical relevance of intracellular forms of *S*. *aureus* are becoming clearer [51], elucidation of the molecular mechanisms of Fn-binding MSCRAMMs, like FnBPA, stands to provide a major contribution towards improving the treatment of staphylococcal infectious diseases.

## Supporting Information

S1 Fig Steady-state fluorescence spectroscopy. Intrinsic tryptophan fluorescence spectra of Fn solutions were obtained on a Spectrofluorimeter LS 50B (Perkin-Elmer) at ambient temperature. (**A**) Fn (0.1 μM) was incubated in TBS in the presence of 1 M GdnHCl, 0.2 μM BBK32 or 2 μM of FnBPA-10. (**B**) FnNTD-30K (1.6 μM) were incubated in TBS in the presence of 16 μM of FnBPA-10. Samples were excited at 295 nm with and excitation slit of 5 nm, and emission spectra were collected with an emission slit of 5 nm. All spectra were corrected for background fluorescence by subtraction of the buffer blanks.
(TIF)

S2 Fig SPR analysis of Fn/α_5_β_1_ interaction. The α_5_β_1_ biosensor was validated by demonstrating metal ion dependence and domain specificity: **(A)** Fn (250 nM) was injected over immobilized α_5_β_1_ surface in the presence of 1 mM MnCl_2_ (solid line) or 3 mM EDTA (dashed line). **(B)** Response curves for a two-fold linear dilution series of Fn-CBD over immobilized α_5_β_1_ are shown. **(C)** Comparison of Fn (250 nM, solid line) and the Fn-NTD fragment lacking the canonical integrin binding RGD-motif (500 nM, dashed line).
(TIFF)
