# Supplementary figures and images for: Allosteric Regulation of Fibronectin/α5β1 Interaction by Fibronectin-Binding MSCRAMMs
Source: PLoS One. 2016 Jul 19;11(7):e0159118. doi: 10.1371/journal.pone.0159118 (PMC4951027; doi:10.1371/journal.pone.0159118)

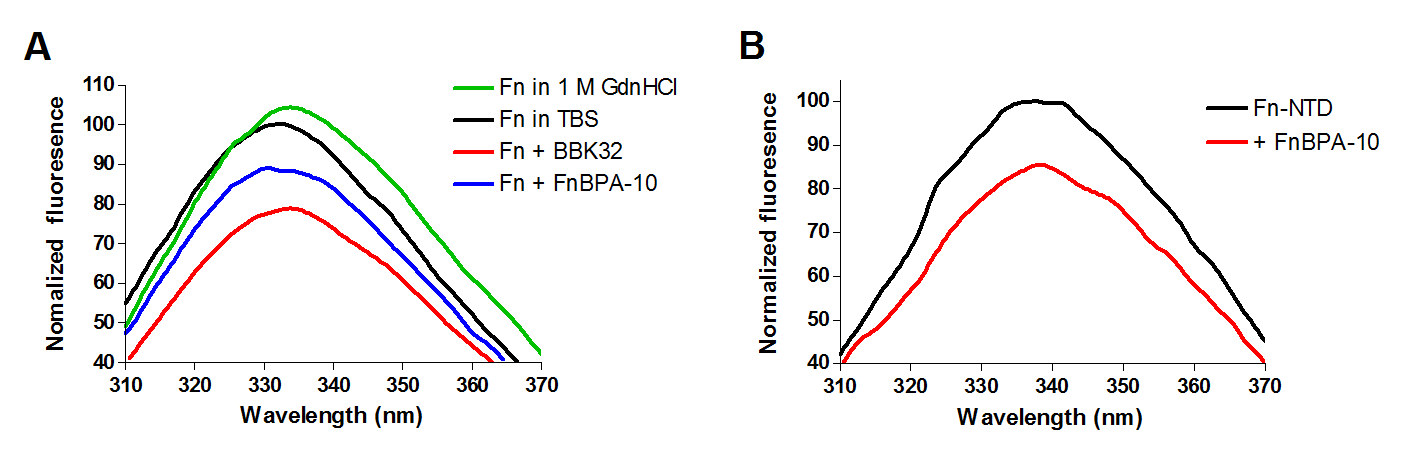

Supplement: S1 Fig — Intrinsic tryptophan fluorescence spectra of Fn solutions were obtained on a Spectrofluorimeter LS 50B (Perkin-Elmer) at ambient temperature. (A) Fn (0.1 μM) was incubated in TBS in the presence of 1 M GdnHCl, 0.2 μM BBK32 or 2 μM of FnBPA-10. (B) FnNTD-30K (1.6 μM) were incubated in TBS in the presence of 16 μM of FnBPA-10. Samples were excited at 295 nm with and excitation slit of 5 nm, and emission spectra were collected with an emission slit of 5 nm. All spectra were corrected for background fluorescence by subtraction of the buffer blanks. (TIF) [file pone.0159118.s001.tif]

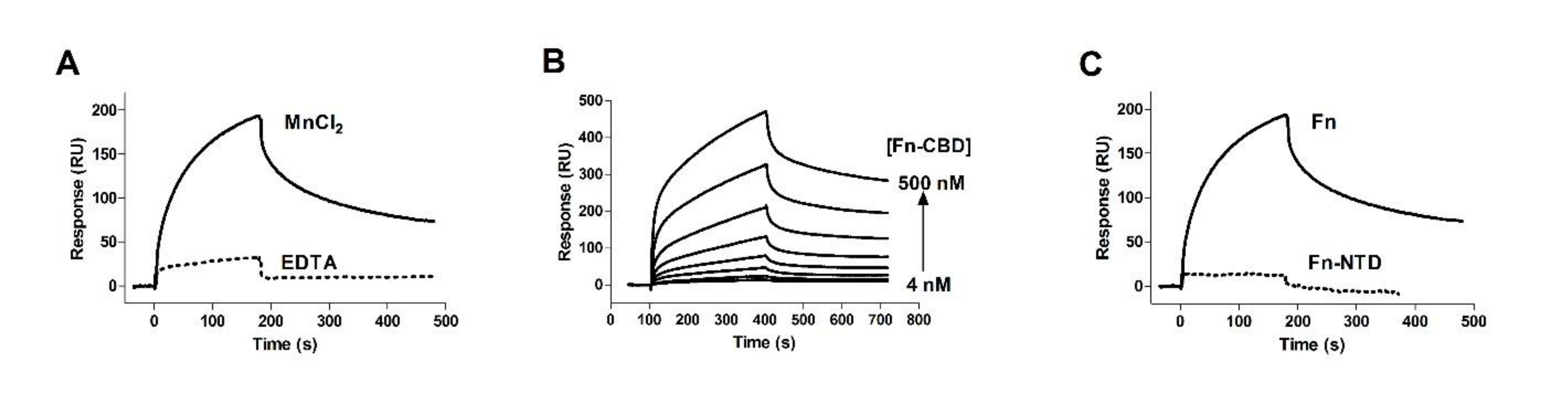

Supplement: S2 Fig — The α5β1 biosensor was validated by demonstrating metal ion dependence and domain specificity: (A) Fn (250 nM) was injected over immobilized α5β1 surface in the presence of 1 mM MnCl2 (solid line) or 3 mM EDTA (dashed line). (B) Response curves for a two-fold linear dilution series of Fn-CBD over immobilized α5β1 are shown. (C) Comparison of Fn (250 nM, solid line) and the Fn-NTD fragment lacking the canonical integrin binding RGD-motif (500 nM, dashed line). (TIFF) [file pone.0159118.s002.tiff]
